# Savannah Medical College, Address of Professor J. G. Thomas

**Published:** 1873-01

**Authors:** 


### *Savannah Medical College—Address of Professor J. G. Thomas.*

We are pleased to learn of the prosperity of the above-named institution. Under its new arrangements and at its new "quarters," the facilities of imparting clinical instruction are much improved. The faculty are hard-working men and accomplished teachers.

Upon the occasion of the opening of the sixteenth session of the institution, Professor Thomas delivered an address worthy of the man and the college he so ably represents. The Savannah Medical College is one Georgia physicians should be proud of. It is deserving of their confidence and support.

